# Factors associated with low childhood immunization coverage among Rohingya refugee parents in Cox’s Bazar, Bangladesh

**DOI:** 10.1371/journal.pone.0283881

**Published:** 2023-04-07

**Authors:** Nawshin Ahmed, A. S. M. Ishtiak, Md. Faisal Kabir Rozars, Atia Sharmin Bonna, K. M. Pritam Alam, Md. Elias Hossan, Rajib Das, Joyeeta Khan, Tahmina Zerin Mishu, Sadia Afrin, Naima Sultana, Md. Reza Al Mamun Rubel, Md. Abdullah Saeed Khan, Nadira Sultana Kakoly

**Affiliations:** 1 North South University, Dhaka, Bangladesh; 2 National Institute of Preventive and Social Medicine, Dhaka, Bangladesh; 3 Chittagong Medical College Hospital, Chittagong, Bangladesh; 4 State University of Bangladesh, Dhaka, Bangladesh; 5 BGC Trust Medical College, Chittagong, Bangladesh; Tokyo Medical and Dental University: Tokyo Ika Shika Daigaku, JAPAN

## Abstract

**Introduction:**

Immunization campaigns and Expanded Program on Immunization (EPI) were launched by Government of Bangladesh (GoB) in collaboration with WHO and other Non-governmental Organizations (NGOs) to tackle the increased risk of vaccine preventable disease outbreak in the Rohingya refugee camps. Immunization coverage was found to be lower than expected. However, a few studies explored the factors behind low vaccine uptake among Refugee children. Therefore, this study was aimed.

**Methods:**

A cross sectional study was carried out among Rohingya parents living in registered camps and makeshift settlements located in Teknaf and Ukhiya upazilla of Cox’s Bazar, Bangladesh. A total of 224 Rohingya parents were conveniently selected (122 parents from each type of camps). Data was collected using a pretested interviewer-administered semi-structured questionnaire with the help of bilingual volunteers who understand Rohingya dialect. All statistical analyses were carried out in IBM SPSS Version 26 (New York, USA).

**Results:**

Total 63.1% of Rohingya parents had good practice regarding childhood immunization (completed EPI vaccination) as per schedule. Of all, 74.6% had good knowledge and 94.7% had positive attitude towards EPI vaccination. Good practice regarding vaccination was significantly more common among parents living in registered camps (77%) than those living in makeshift settlements (49.2%, p<0.001). Multivariable logistic regression analysis revealed that living in registered camps (Adjusted Odds Ratio [aOR]: 2.99; 95% Confidence Interval [CI]: 1.41–6.32) and good knowledge level (aOR: 2.88; 95%CI: 1.32–15.82) were independent determinants of good practice. A separate analysis in both type of camps revealed that in registered camps, good knowledge level (aOR: 3.62; 95%CI: 1.45–9.04) and having >2 children (aOR: 3.71; 95%CI: 1.34–10.27), and in makeshift settlements, father’s employment (aOR: 2.33; 95%CI: 1.34–6.72), father’s education (aOR: 3.00; 95%CI: 1.34–6.72) and presence of any electronic device (e.g., radio, television, mobile phone) (aOR: 4.01; 95%CI: 0.96–16.84) were significant determinants of good childhood immunization practice.

**Conclusion:**

Health education and promotion strategies should be implemented to increase knowledge and awareness about EPI immunization benefits among Rohingya parents to ensure greater coverage.

## Introduction

The Expanded Programme on Immunization (EPI), introduced by the World Health Organization (WHO) in 1977 in Alma-Ata, is the most successful global public health program saving millions of lives of children every year [1]. Through vaccination, smallpox was eradicated, polio is on the verge of eradication, and neonatal tetanus incidence was reduced by 88%. The incidence of measles, rubella, diphtheria, pertussis, childhood TB, and hepatitis B is considerably low because of the implementation of EPI in 192 member states of WHO [2]. The recent addition of vaccines against human papillomavirus, rotavirus, yellow fever virus, pneumococcus, and *Hemophilus influenzae* in the EPI in different countries has been instrumental in reducing the burden of these diseases. Bangladesh launched EPI in 1979 and covered as much as 94.6% of live births in 2020 [3]. For its sustained high coverage and contribution in reducing child mortality, Bangladesh has been appreciated and awarded multiple times [1].

In 2017, a complex humanitarian crisis arose when Rohingya people living in Rakhaine state of Myanmar were forcibly displaced from their country through systematic human rights violations and military crackdown [4], and more than seven hundred thousand Rohingya refugees arrived to join two hundred thousand refugees already living in the camps in Cox’s Bazar, Bangladesh [2]. The makeshift settlements resided by the newly arrived refugees became overcrowded with a food shortage and a lack of access to health services. A public health emergency from infectious disease was imminent, with low vaccination coverage and malnutrition among the Rohingyas [5, 6]. Measles and diphtheria outbreak ensued in the Rohingya camps in between September and November 2017 and started to spread rapidly across camps [6–8]. However, the Government of the People’s Republic of Bangladesh (GoB) promptly responded to the crisis by starting mass vaccination campaigns to immunize Rohingya children against diphtheria, measles and other vaccine preventable diseases with the aid of global public health partners and vaccine alliances [8]. In addition, routine immunization through EPI was promoted, particularly by the voluntary health workers, to ensure wider coverage and sustain vaccination activities among the refugees [9, 10].

Studies conducted among different refugee and migrant population around the world identified a low vaccination rate, and various associated factors, including—duration of residence in the host country, citizenship status, country of origin, language, disease perceptions, belief & culture, literacy, knowledge, motivation, employment, socio-economic status, and health care & insurance provision [11–16]. One vaccination coverage and seroprevalence survey conducted [2] after immunization campaigns among the Rohingya community found that approximately 10 to 40% of children did not have seroprotection against measles, rubella, diphtheria, and tetanus, implying an inadequate coverage or non-response. Moreover, absence of seroprotection was significantly higher among children living in makeshift settlements compared to that of registered camps. However, a few studies explored the determinants of low vaccination rate among Rohingya children residing in Bangladesh. Therefore, the present study aimed to assess the practice of childhood immunization and associated factors among Rohingya parents to explore the reasons for the gap remaining in EPI coverage.

## Methods

### Study place, population and period

This cross-sectional study was carried out among Rohingya parents living in Teknaf and Ukhiya upazilla (level 3 administrative area) of Cox’s Bazar, Bangladesh between December 2021 and January 2022. A total of 244 Rohingya parents with at least one child aged 7 years were included. Data was collected from two registered camps (Kutupalong Refugee Camp [KRC] and Nayapara Refugee Camp [NRC]) and two unregistered camps/makeshift settlements (Camp no 7 and Camp no 26) to ensure enrolment of Refugees who arrived before 2017 and who were forcibly displaced from Rakhaine state of Myanmar during the 2017 massacre, respectively [17]. According to Bangladesh’s Refugee Relief and Repatriation Commission (RRRC) and United Nations High Commissioner for Refugees (UNHCR) family counting data [18] KRC, NRC, camp 7 and camp 26 had 3129, 4263, 8313 and 3328 families, respectively. The following formula was used for sample size calculation n = Z^2^p(1-p)/d^2^. Due to unavailability of overall vaccination coverage data, taking the prevalence of BCG vaccination (p = 84%) from Feldstein [2] and considering a standard normal deviate at 95% confidence interval (Z = 1.96) and an effect size d of 5%, the sample size was calculated to be 206. Adjusting for 20% presumed non-response the sample size came out to be 247.8 (~248). Hence, we initially planned to divide the sample equally and select 124 parents from each type of camps (i.e., from registered camps and makeshift settlements). However, 122 parents could be included within the data collection period. Parents who were living outside registered camps and makeshift settlements, who were unable or unwilling to give information on vaccination of their children were excluded from the study.

### Data collection instrument

A pretested interviewer administered semi-structured questionnaire was used for data collection. The questionnaire comprised two sections. The first section asked about sociodemographic profile of parents including age, sex, occupation of the respondent and spouse, education level of the respondent and spouse, monthly family income, electronic media device (radio, television, mobile phone) in possession, number of household members (living in the same household), number of children ever born, age of young and old children, number of rooms in the house, any child lost (died) due to disease or other causes, and any child every admitted in the hospital due to disease or other cause. The second section comprised of knowledge, attitude and practice related questions. This section covered the following areas. If the respondent ever attended any awareness raising programs, if his/her child received EPI vaccines as per schedule, status of their knowledge of benefits and side-effects of vaccination, and their attitude towards childhood immunization. The knowledge questions carried three options- yes, no and don’t know, and the attitude section carried three-point Likert responses which varied based on the questions asked (Please see S1 File for details). The practice related question had three options–yes, no and don’t know (i.e., unsure). Practice related questions asked about vaccination of children as per EPI schedule (this was ensured by checking the EPI card), whether they sought vaccines other than those enlisted in EPI for their children and ways of managing minor side effect of vaccination.

### Data collection procedure

Data was collected by a group of trained local community health workers who understood both Bangla and Rohingya language. The questionnaire was translated from English to Bangla for training and data collection by an investigator who is proficient in both languages. After formal permission from local authorities to approach the selected camps, the data collectors approached and explained the study purpose to conveniently selected Rohingya parents based on the inclusion and exclusion criteria. Then they asked for their consent to participate in the study. Those who gave consent was further interviewed face to face. They were given the option to withdraw from the interview at any time. For any difficulty in understanding the questions were repeated and explained using appropriate words.

### Scoring of knowledge, attitude and practice related question

A total of 10 knowledge related questions and 4 attitude related questions were considered for scoring after reliability analysis. The Cronbach’s alpha (α) value of the final set of knowledge and attitude questions were 0.726 and 0.614 respectively. The only practice related questions retained in the analysis was the question asking practice regarding childhood EPI vaccination which was confirmed by checking their vaccination card. Respondent who completed vaccination according EPI schedule appropriate for their children were considered to have good childhood immunization practice. Incomplete vaccination or no vaccination was considered poor practice. Other practice related questions were excluded from analysis due the unobserved subjective nature of responses. A detailed scoring scheme used for each question is enlisted in S1 Table. The total knowledge and attitude score ranged between 0 to 10 and 1 to 12, respectively. The total scores were categorized using Bloom’s cut-off point of 80%. A total knowledge score of ≥8 was considered good, otherwise considered poor knowledge. A total attitude score of ≥10 was considered positive, otherwise considered negative attitude.

### Outcome and explanatory variables

Practice of vaccination, as confirmed by asking the parents and cross-checking vaccination card, was considered as outcome variable. Explanatory variables having potential association with practice was selected by reviewing previous published studies on migrant and refugees [2, 13, 14] and after discussion with experts. Study context and constraints were also taken into consideration. All of the factors tested against vaccination practice in the bivariate analysis including- camp type, age, sex, fathers’ occupation & education, mother’s education, monthly family income, presence of any electronic media, number of children, any children lost or admitted in the hospital, family size, knowledge and attitude level regarding immunization were considered for inclusion in the multivariable analysis except mother’s occupation. Occupation of mother was excluded because categories other than housewives had very low frequency of response.

### Statistical analysis

Data was entered, curated and analyzed using IBM SPSS Statistics Version 26 (New York, USA). Descriptive statistics were expressed as frequency (percentage) and mean (±standard deviation [SD]) for categorical and continuous data, respectively. The independent sample t test and chi-square test was used for bivariate analysis. Univariate and multivariable logistic regression analysis was used for exploring factors associated with good childhood immunization practice. A p-value of <0.05 was considered significant for all statistical tests.

### Ethical consideration

The study was reviewed and approved by the institutional review board of North South University (No– 2021/OR-NSU/IRB/1101). Formal permissions were taken from the Office of the Refugee Relief and Repatriation Commissioner, Cox’s Bazar of Government of the People’s Republic of Bangladesh for data collection in the selected Rohingya Camps. All procedures were conducted according to the guidelines of the Declarations of Helsinki. Informed consent was taken from the parents before participation in the study.

## Result

**Fig 1:**
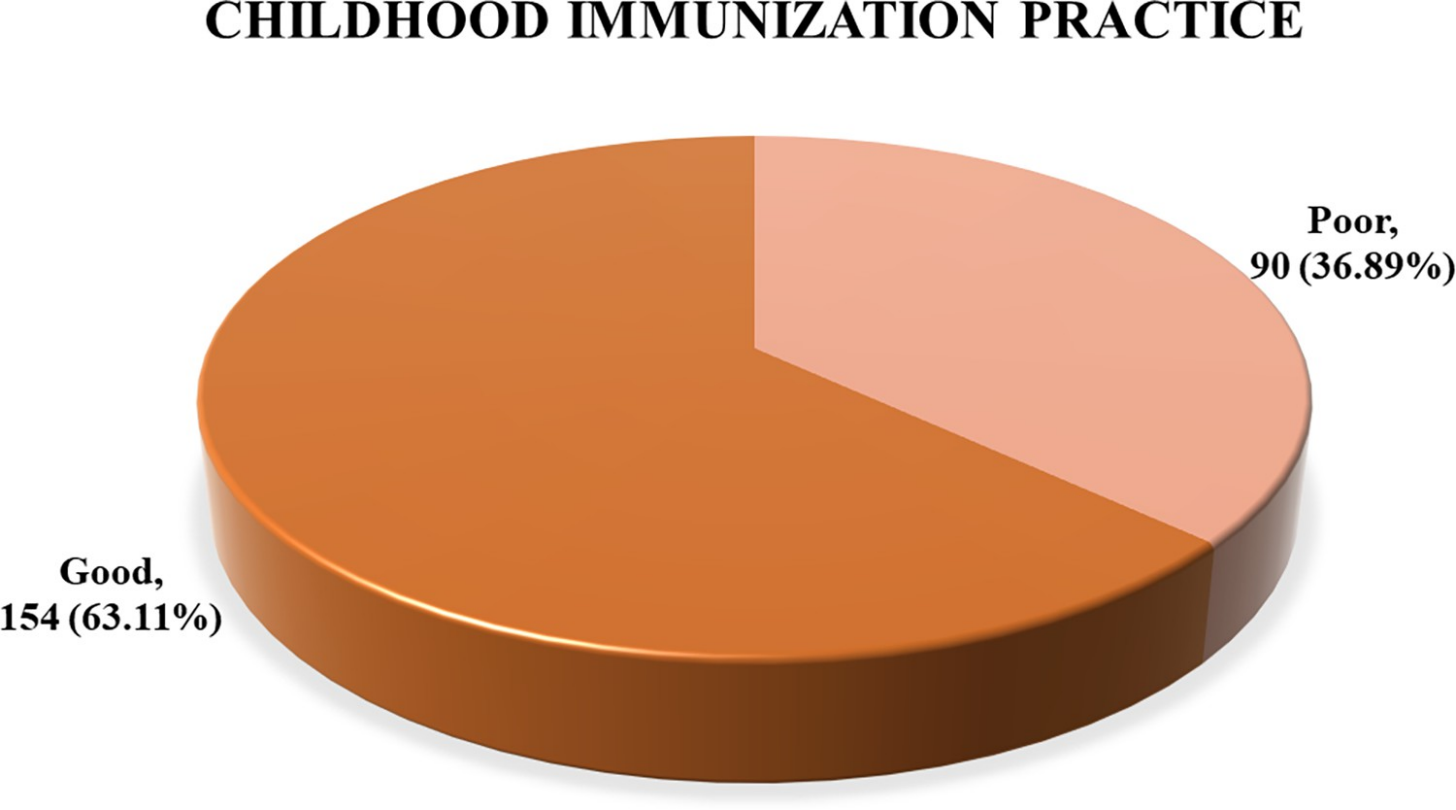
Childhood immunization practice pattern among Rohingya refugee parents.

A total of 63.1% (n = 154) of parents had good practice regarding vaccination of their children (completed EPI vaccination as per schedule) (Fig 1).

The average age of the parents was 28.9 ± 7.4 years (±SD). Of all, 50.8% were female, and 49.2% were male. There was no difference in vaccination practice in relation to age and sex. Significantly more Rohingya parents (77%) staying at registered camps had good practices regarding EPI vaccination than FDMN parents (49.2%) staying at makeshift settlements (p<0.001). Among all respondents, 57.4% of mothers and 46.7% of fathers did not have any formal education. The vaccination practice was significantly lower among uneducated parents (p = 0.025 for mothers and p = 0.001 for fathers). Ninety-one percent of mothers were housewives. While the majority of fathers were day laborers (35.2%), and 20.9% of them did not have any job. Vaccination practice was significantly higher in proportion among those fathers who had jobs than those who did not have a job (p = 0.008). A significantly higher proportion of respondents with a monthly family income of ≥6000BDT had good vaccination practice (p = 0.016). Ninety-one percent of respondents had an electronic device, and 44.3% of families had >2 children. These factors were not associated with practice. Those who lost at least one of their children were significantly more likely to practice vaccination than those who did not fail (p = 0.009). Sixteen percent of respondents’ children had a previous history of admission, 48.4% had a large family (>4 members), 93.0% attended immunization session(s), 74.6% had good knowledge, and 94.7% had a positive attitude towards EPI vaccination. None of these characteristics showed a significant association with EPI vaccination practice (p>0.05 for all) (Table 1). More details on responses to the knowledge and attitude-related questions are available in S2 Table.

**Table 1:** Participant characteristics in respect to childhood immunization practice of Rohingya parents.

Variable	EPI vaccination practice		*p-value*	Total
	Poor (n = 90)	Good (n = 154)		n (%)
Age (years), mean ±SD	29.4 ±7.8	28.2 ±6.5	0.242	28.9 ±7.4
Sex, n (%)				
Male	45 (37.5)	75 (62.5)	0.845	120 (49.2)
Female	45 (36.3)	79 (63.7)		124 (50.8)
Camp type, n (%)				
Registered	28 (23.0)	94 (77.0)	<0.001	122 (50.0)
Makeshift settlements	62 (50.8)	60 (49.2)		122 (50.0)
Mother’s education, n (%)				
No education	60 (42.9)	80 (57.1)	0.025	140 (57.4)
Primary and above	30 (28.8)	74 (71.2)		104 (42.6)
Father’s education, n (%)				
No education	54 (47.4)	60 (52.6)	0.001	114 (46.7)
Primary and above	36 (27.7)	94 (72.3)		130 (53.3)
Mother’s occupation, n (%)				
House wife	81 (36.5)	141 (63.5)	0.083	222 (91.0)
Service	3 (50.0)	3 (50.0)		6 (2.5)
Others	1 (11.1)	8 (88.9)		9 (3.7)
None	5 (71.4)	2 (28.6)		7 (2.9)
Father’s occupation, n (%)				
Day labor	35 (40.7)	51 (59.3)	0.008	86 (35.2)
Service	11 (33.3)	22 (66.7)		33 (13.5)
Fisherman	2 (2.2)	15 (9.7)		17 (7.0)
Others	15 (26.3)	42 (73.7)		57 (23.4)
No job	27 (52.9)	24 (47.1)		51 (20.9)
Monthly Family income (BDT), n (%)				
<6000	69 (42.1)	95 (57.9)	0.016	164 (67.2)
≥6000	21 (26.3)	59 (73.8)		80 (32.8)
Any electronic media, n (%)				
Present	79 (35.6)	143 (64.4)	0.181	222 (91.0)
Absent	11 (50.0)	11 (50.0)		22 (9.0)
Number of children, n (%)				
≤ 2	53 (39.0)	83 (61.0)	0.449	136 (55.7)
> 2	37 (34.3)	71 (65.7)		108 (44.3)
Any children lost, n (%)				
Yes	3 (12.5)	21 (87.5)	0.009	24 (9.8)
No	87 (39.5)	133 (60.5)		220 (90.2)
Any children ever admitted in hospital, n (%)				
Yes	10 (25.6)	29 (74.4)	0.112	39 (16.0)
No	80 (39.0)	125 (61.0)		205 (84.0)
Family size (members), n (%)				
Small (≤4)	48 (38.1)	78 (61.9)	0.686	126 (51.6)
Large (>4)	42 (35.6)	76 (64.4)		118 (48.4)
Attended immunization session, n (%)				
Yes	83 (36.6)	144 (63.4)	0.704	227 (93.0)
No	7 (41.2)	10 (58.8)		17 (7.0)
Knowledge level, n (%)				
Good	64 (35.2)	118 (64.8)	0.340	182 (74.6)
Poor	26 (41.9)	36 (58.1)		62 (25.4)
Attitude level, n (%)				
Positive	85 (36.8)	146 (63.2)	0.904	231 (94.7)
Negative	5 (38.5)	8 (61.5)		13 (5.3)

P-value was determined by independent samples t test, Fisher’s exact test, and chi-square test where appropriate. Significant p-values are shown in bold.

Table 2 enlists the output of univariate and multivariable logistic regression analysis carried out to assess factors associated with good childhood immunization practice among Rohingya parents. On univariate analysis, registered camp (cOR: 3.46; 95%CI: 1.99–6.02), employed father (cOR: 2.32; 95%CI: 1.24–4.35), educated mother (cOR: 1.85; 95%CI: 1.38–3.99), monthly family income of ≥6000 BDT (cOR: 2.04; 95%CI: 1.13–3.67), and any children lost (cOR:4.58; 95%CI: 1.32–15.82) were significant determinants of good immunization practice. On multivariable analysis, after adjustment for other factors, registered camp (aOR: 2.99; 95%CI: 1.41–6.32), and good knowledge level (aOR: 2.88; 95%CI: 1.32–15.82) remained as significant determinants of practice.

**Table 2:** Logistic regression analysis to assess factors associated with good childhood immunization practice among Rohingya parents.

Variables	Reference	cOR (95%CI)	*p-value*	aOR^$^ (95%CI)	*p-value*
Camp type (Registered)	Makeshift settlements	3.46 (1.99–6.02)	<0.001	2.99 (1.41–6.32)	0.004
Age (years)		1.02 (0.99–1.06)	0.242	1.01 (0.97–1.06)	0.548
Sex (Male)	Female	0.95 (0.56–1.60)	0.845	1.26 (0.62–2.56)	0.527
Father’s occupation (Employed)	Unemployed	2.32 (1.24–4.35)	0.008	1.65 (0.84–3.24)	0.143
Mother’s education (Primary and above)	No education	1.85 (1.08–3.18)	0.026	1.24 (0.60–2.55)	0.558
Father’s education (Primary and above)	No education	2.35 (1.38–3.99)	0.002	1.65 (0.84–3.24)	0.143
Monthly family income in BDT (≥6000)	<6000	2.04 (1.13–3.67)	0.017	1.24 (0.54–2.85)	0.620
Any electronic media (Present)	Absent	1.81 (0.75–4.36)	0.186	1.18 (0.43–3.28)	0.748
Number of children (>2)	≤2	1.22 (0.72–2.07)	0.449	1.87 (0.63–5.58)	0.257
Any children lost (Yes)	No	4.58 (1.32–15.82)	0.016	1.53 (0.67–3.67)	0.344
Any children ever admitted in hospital (Yes)	No	1.86 (0.86–4.02)	0.116	1.53 (0.64–3.67)	0.344
Family size in members (>4)	≤4	1.11 (0.66–1.87)	0.686	0.56 (0.20–1.61)	0.281
Attended immunization session (Yes)	No	1.21 (0.44–3.31)	0.704	1.04 (0.35–3.08)	0.948
Knowledge level (Good)	Poor	1.33 (0.74–2.40)	0.341	2.88 (1.30–6.41)	0.009
Attitude level (Positive)	Negative	1.07 (0.34–3.39)	0.904	0.75 (0.18–3.06)	0.684

p-values are shown in bold; cOR: Crude Odds Ratio; aOR: Adjusted Odds Ratio

^$^Enter method was used for adjustment.

Multivariable logistic regression analysis separated by two types of camps showed that good knowledge level (aOR: 3.62; 95%CI: 1.45–9.04) and >2 children (aOR: 3.71; 95%CI: 1.34–10.27) were independent determinants of good practice in registered camps. While father’s employment (aOR: 2.33; 95%CI: 1.34–6.72), father’s education (aOR: 3.00; 95%CI: 1.34–6.72) and presence of any electronic device (e.g., radio, television, mobile phone) (aOR: 4.01; 95%CI: 0.96–16.84) were significant factors associated with good childhood immunization practice among makeshift settlements (Table 3).

**Table 3:** Multivariable logistic regression analysis to assess factors associated with good childhood immunization practice among Rohingya parents staying at registered and temporary camps.

Variables	Reference	aOR (95%CI)^$^	*p-value*
Registered camps*			
Knowledge level (Good)	Poor	3.62 (1.45–9.04)	0.006
Number of children (>2)	≤2	3.71 (1.34–10.27)	0.012
Makeshift settlements**			
Father’s occupation (Employed)	Unemployed	2.33 (1.03–5.28)	0.044
Father’s education (Primary and above)	No education	3.00 (1.34–6.72)	0.008
Any electronic media (Present)	Absent	4.01 (0.96–16.84)	0.057

^$^A stepwise forward LR method was used.

*After adjustment for participant’s age, sex, mother’s education, father’s education, father’s occupation, monthly family income, family size, presence of electronic device, children lost, children previously admitted, and attitude regarding EPI vaccination.

**After adjustment for participant’s age, sex, mother’s education, monthly family income, family size, number of children, children lost, children previously admitted, knowledge and attitude regarding EPI vaccination.

## Discussion

There has been a substantial reduction of child mortality in the world after the introduction and implementation of EPI by WHO [19]. However, humanitarian emergencies often break down regular health service including routine vaccination programs. Consequently, displaced population are destined to live in crowded camps with poor water and sanitation, food shortage, and poor healthcare access, placing them at a high risk of morbidity and mortality due to outbreaks of vaccine preventable diseases [20]. The Rohingya ethnic minorities had been systematically deprived of human rights [4] and the EPI coverage in this population had already been low before fleeing to Bangladesh [21]. Moreover, measles and diphtheria cases broke out in the camps at the end of 2017 [6–8]. Considering all these factors, the Government of Bangladesh, with aid from WHO and other agencies, directed several mass vaccination-campaigns alongside the routine vaccine delivery through EPI.

We explored the current childhood immunization practice among Rohingya refugee parents as per EPI schedule as well as the determinants of good practice. Our analysis revealed that nearly two-third of Rohingya respondents immunized their children according to EPI schedule. This is higher than that previously estimated among Refugees in Kutupalong camps [22] where the vaccine uptake for different diseases ranged between 23% to 78.7%. We also found that parents living in the registered camps were significantly more likely to be vaccinating their children than those of makeshift settlements. Our finding is concordant with previous reports where recently arrived (i.e., during and after 2017) Rohingya refugees were reported to have lower vaccination rates than those who arrived earlier [2, 22]. In addition, the recently arrived refugees showed a lower coverage even after the mass immunization campaigns [2]. This phenomenon seems to be common among the refugee and migrant population throughout the world. Crawshaw *et al* [12] found that being a recent migrant or having a refugee status was associated with lower vaccination rates in European countries. Charania *et el* [13] reviewed the immunization coverage among migrant and non-migrant people around the world and found that the duration of residence in the host country is an important factor associated with vaccine uptake among the migrants. Newly arrived refugees take time to settle in and assimilate in the new habitat, while the host government and international agencies come forward to tackle the crisis. This probably explain the gap in vaccination coverage between old and new refugees. The Rohingya refugees living in the registered camps had been residing here since long before 2017. Hence, it is expected that they were more adapted to their new environment and socialized with the host community. Moreover, it is likely that they were aware about and taking service from routine vaccination programs from local health facilities, explaining why parents from these camps in our study were more likely have good childhood immunization practice.

There are several other determinants of vaccine coverage among Rohingya refugees. On bivariate analysis, in addition to camp type, we found several other significant determinants of vaccine coverage among the refugee children. Mother and father’s education, father’s occupation, monthly family income, and previous loss of children were discovered to be associated with good vaccination practice. Our findings are congruent with that of Debela, Garrett and Charania [23] who studied the determinants of vaccine hesitancy among resettled refugee parents in New Zealand and found that parents’ education were significantly associated with vaccine hesitancy. Moreover, mother or caretaker education appears to be important predictor of vaccine acceptance even in the native citizens of Bangladesh [24, 25], and of low and middle-income countries around the world [26]. Generally, economic status of mother or caretaker is considered an important determinant of vaccination coverage, which determines their ability to transport children to health facility and, avail vaccines where it is not freely available or covered by insurance [26]. However, as Rohingya refugees were mostly living on humanitarian aids and majority of the health facilities were within their reach, respondents’ monthly family income, rather than directly influencing their vaccination, might have indirectly influenced vaccination practice by affecting their awareness or knowledge. This also becomes obvious when the results of multivariable adjustments are taken into account. We found that living in registered camps and presence of good knowledge were independently associated with good immunization practice when adjusted for other factors. Which means parent’s education, father’s occupation and monthly income were reflected in their knowledge about the benefit of vaccination in the study respondents. Phillips *et al* [26] listed awareness as an important determinant of vaccination coverage in low and middle-income countries emphasizing the importance of it in determining the vaccine acceptance and, hence, coverage.

Noticeably an experience of loss of any children were found to associated with good practice. Loss of child is a significant life event for any parents, which could have brought about realization of the importance of protecting lives of the rest of their child by available means.

We carried out separate multivariable analysis for registered camps and makeshift settlements to understand the separate influence of factors influencing vaccination practice in these two areas. Our analysis revealed that knowledge level of parents and having more than 2 children were the determinants of good practice in registered camps. Which goes along our hypothesis that the past experience of living in the registered camps might have influence practice regarding childhood immunization. On the other hand, practice of vaccination among those newly arrived refugees settling in makeshift shelters were independently influenced by father’s occupation, father’s education and presence of any media devices. As Rohingya society is traditionally patriarchal [27], which means fathers play decision making role in the family. Therefore, father’s education is important in imparting awareness regarding importance of vaccine in preventing child deaths. Also, employed father are more likely to meet family needs. Presence of electronic device like TV, radio or mobile phone ensures that people get to know about the available vaccination services. Feldstein *et al* noted that vaccination campaigns among Rohingya refugees in 2017 could not reach some families because they were unaware about the campaign [2], indicating the importance of awareness about the health services in the target population.

### Strengths and limitations

The present study was limited in that it used convenient sampling to select respondents. Hence, the findings of this study might not be generalizable, and the proportion of good practice regarding vaccination doesn’t represent the prevalence of complete vaccination in the Rohingya community. Also, health service-related factors were not elicited in this study, leaving potential determinants unexplored. Another important limitation was a low reliability statistic of final attitude question set. Hence, the attitude score determined in our respondents might not have properly reflected their health beliefs and should be read within context. Despite the limitation, the study draws a picture of the current childhood immunization situation and sheds lights on some important factors behind immunity gaps among Rohingya refugees living in registered and unregistered camps in Bangladesh. Lack of information, knowledge and education regarding vaccination, and unemployment were discovered to be the main determinants of inadequate vaccination of children in Rohingya community.

### Implications for policy and practice

Our findings indicate that there is a general lack of awareness regarding the importance of vaccination among Rohingya refugees, especially among those who were forcibly displaced in recent years and were living in makeshift settlements. Lower education level, lack employment and inadequate access to mass media could be some of the factors behind low awareness. Therefore, policymakers should emphasize on health education and promotion of Rohingya community to ensure that target level of vaccination is achieved.

## Conclusion

The Rohingya ethnic minority went through a dire humanitarian crisis requiring external assistance to survive and thrive. Government of Bangladesh responded to the crisis with the utmost humane view and received hundreds of thousands of forcibly displace Rohingya people and gave them shelter, food, water and health services with assistance from WHO and other national and international NGOs. Any mass population displacement can increase the risk of vaccine-preventable disease outbreaks because of the limitation of health service, poor living conditions and malnutrition. Hence, mass vaccination campaigns are run in addition to strengthening of routine immunization services. This study found that vaccination coverage was low, particularly among those refugees living in unregistered camps, and those with poor knowledge about vaccination. Increasing knowledge about the importance of vaccination in reducing childhood morbidity and mortality is required to increase acceptance of vaccine among Rohingya parents. Therefore, we recommend taking necessary community-based strategy for increasing awareness among Rohingya communities to ensure proper vaccination of their children.

## Supporting information

S1 File Questionnaire. (DOCX)

S1 Table Knowledge, attitude and practice question scoring scheme. (DOCX)

S2 Table Distribution of responses to knowledge (K), attitude (A) and practice (P) related questions. (DOCX)
