# Supplementary material for: Factors associated with low childhood immunization coverage among Rohingya refugee parents in Cox’s Bazar, Bangladesh
Source: PLoS One. 2023 Apr 7;18(4):e0283881. doi: 10.1371/journal.pone.0283881 (PMC10081790; doi:10.1371/journal.pone.0283881)
Supplement: S1 File — (DOCX) [file pone.0283881.s001.docx]

**A comparative cross-sectional study to assess knowledge, attitude and practices towards EPI vaccination between Rohingya parents who are living in the registered camps versus new makeshift settlements in Cox’s Bazar, Bangladesh.**

General objective:

A KAP survey can generate data that can be used for the following purposes:

To identify knowledge gaps, cultural beliefs, and behavioral patterns regarding EPI vaccination among the Rohingya parents for vaccinating their child/children that may identify their needs, problems, and barriers for vaccinating their child/children aged from 0-7 years old to help in planning and implement interventions in the Rohingya camps in Cox’s Bazar. To deepen the understanding of commonly known information, assessing attitudes, and factors that influence behavior towards EPI vaccination. To assess and identify communications processes and sources important for program implementation and effectiveness of EPI vaccination at Rohingya Camps in Cox’s Bazar, Bangladesh.

Specific objectives: (To assess the practice will refer to quantitative study and to explore the believes will refer to qualitative study)

* To describe the socio demographic factors and socio-economic factors of Rohingya parents who have children aged from 0-7 years old (i.e child/children from newborn to 7 years old)

* To identify the knowledge of the Rohingya parents about the immunization programs and childhood EPI vaccination.

* To assess the attitude of the Rohingya parents towards EPI vaccination and immunization programs for 0-7 years aged children.

* To explore the practice/beliefs of the Rohingya parents about EPI vaccination and immunization program in Rohingya refugee camps in Cox’s Bazar, Bangladesh.

**Annexes:**

**Section one:**

**Child’s parents’ information:**

| **Part. 1** | | | | |
| --- | --- | --- | --- | --- |
| 1. Serial Number/ MRC or FCN number | 2. Name: | 3. Age: | Gender:   1. Male 2. Female | 5. Occupation:  1. Day labour  2. Businessman  3. Teacher  4. Service  5. Maid Servant  6. Tailor  7. Shop Keeper  8. Mechanic  9. Cook  10. Volunteer  11. House Wife  12. Car driver  13. No Job  14. Fisher Man |
| 6. Education level of Mother from:   - - - - Myanmar or       - Bangladesh | | 1. No Education 2. Primary 3. Secondary 4. Higher secondary | | |
| 7. Education level of Father from:   - - - - Myanmar or       - Bangladesh | | 1. No education 2. Primary 3. Secondary 4. Higher secondary | | |
| 8. What best describes your spouse’s job? | |  | | |
| 09. Where in the following range does your household  monthly income fits? | | 1. Below 6,000 2. 6,000 – 10,000 3. 10,000 - 20,000 4. 20,000 and above 5. No income but they get relief | | |
| 10. Which of the belongings you have at home from the list? Please circle each thing if you have more than one. | | 1. Radio  2. Television  3. Mobile phone  4. More than one item  5. No Electronic device | | |
| 11. How many members are in the household (also mention their ages)? | | Total Number : | | |
| 12. Number of children you gave birth to? | |  | | |
| 13. What is the age of your oldest child? | |  | | |
| 14. What is the age of your youngest child? | |  | | |
| 15. How many rooms do you have at your house? | |  | | |
| 16. Have you lost any of your child regarding any illness? | | 1. Yes 2. No | | |
| 17. If Yes, then do you remember the disease? | | 1. Did not loss 2. Pneumonia 3. Diarrhoea 4. Measles 5. Cholera 6. Diphtheria 7. Tetanus 8. Other: ........ 9. don’t know the disease 10. don’t remember | | |
| 18. Has any of your child ever admitted to hospital due to any infectious diseases? | | 1. Yes  2.No | | |
| 19. If yes, then please also mention the name of the disease if you know . | | 1. Communicable disease- Pneumonia, Diarrhoea, Measles, Cholera, Diphtheria, Tetanus, Enteric fever. 2. Non-communicable disease- Asthma, Congenital disease, bronchiolitis, electrolyte imbalance, too much headache. 3. Don’t know the disease. 4. Patient forgot the disease 5. No. | | |

**Part 2: Knowledge (K), attitude (A) and practice (P) related questions and awareness session attendance:**

| 20.  a. Have you attended immunization awareness session?    b. Have you attended any sanitation and hygiene awareness session?  c. Have you attended any breast-feeding awareness session?  d. Have you attended any hand  washing awareness session? | 1. YES 2. NO 3. YES 4. NO 5. YES 6. NO 7. YES 8. NO |
| --- | --- |
| 21. How old was your child at the time he/she received first vaccine shot? (Need to check with vaccine card). (P) | 1. 0 – 4 weeks (after birth)  2. 5 - 9 weeks  3. 6-14 weeks  4. 9- 15 months  5. 15 18 months  6. 2 years  7. 1 Year – 5 years  8. 5 – 7 years  9. Did not vaccinate |
| 22. Where do you immunize your child?  If the answer was “At home” what are the main concerns that make you not to visit health facilities? (P) | 1. Health facility 2. At home 3. Missed immunization 4. Others (Please mention) such as Myanmar schools 5. Hospital |
| 23. Information on whether the child/children received EPI scheduled vaccines timely (confirmed by seeing the vaccination card from the participant)? (P)  List of EPI scheduled vaccines:   1. OCV (1. Yes 2. No) 2. BCG Birth Dose (1. Yes 2. No) 3. Rotavirus (1. Yes 2. No) 4. Hepatitis Birth Dose (1. Yes 2. No) 5. Oral Poliovirus Vaccine   (1. Yes 2. No)   1. Pentavalent Vaccine (1. Yes 2. No) 2. Pneumococcal Pneumonia (conjugate)   (1. Yes 2. No)   1. Measles Containing Vaccine (Anti Measles, Mumps, Rubella) 2. Yes 2. No) 3. Tetanus Toxoid (1. Yes 2. No) | 1. Yes 2. No |
| 24. Do you know that immunization is beneficial for health? (K) | 1. Yes 2. No 3. Don’t know |
| 25.Do you know that immunizations can protect my child from communicable diseases. (K) | 1. Yes 2. No 3. Don’t know |
| 26. It is important to immunize my children on schedule? (A) | 1. Agree 2. Neither agree nor disagree 3. Disagree |
| 27. Do you know that some vaccines are associated with fever and pain. (K) | 1. Yes 2. No 3. Don’t know |
| 28. Do you know that vaccination could result in skin rash. (K) | 1. Yes 2. No 3. Don’t know |
| 29. Have you given your children the obligatory vaccines (P) | 1. Yes  2. No  3. I don’t know |
| 30. Do you know that vaccination is very important for children from the first day of birth (K) | 1. Yes  2. No  3. I don’t know |
| 31. Do you know that vaccination prevent infectious disease (K) | 1. Yes  2. No  3. I don’t know |
| 32. Do you know that vaccination decreases the rates of mortality and disabilities (K) | 1. Yes  2. No  3. I don’t know |
| 33. Do you know that vaccination could maintain child health (K) | 1. Yes  2. No  3. I don’t know |
| 34. Do you know that childhood vaccines could control Measles (K) | 1. Yes  2. No  3. I don’t know |
| 35. Do you know that hepatitis B virus could be prevented by vaccination (K) | 1. Yes  2. No  3. I don’t know |
| 36. Do you know that diphtheria, tetanus and pertussis could be controlled by vaccination (K) | 1. Yes  2. No  3. I don’t know |
| 37. Do you know that malnutrition, low fever and diarrhea are not contraindications for vaccination (K) | 1. Yes  2. No  3. I don’t know |
| 38. Do you know that even healthy child needs vaccination (K) | 1. Yes  2. No  3. I don’t know |
| 39. What do you think about vaccination benefits? (A) | 1. Beneficial  2. Not beneficial  3. I don’t know |
| 40. What do you feel when vaccinating your child? (A) | 1. Safe  2. Fear  3. I don’t know |
| 41. Are you in favor of obligatory vaccination programs designed by the health authorities? (A) | 1. Yes  2. No  3. I don’t know |
| 42. Will you give advice your relatives and family to immunize their children? (A) | 1. Yes  2. No  3. I don’t know |
| 43. Do you follow the obligatory vaccination programs? (P) | 1. Yes  2. No  3. I don’t know |
| 44. Do you search for other available vaccines for your children? (P) | 1. Yes  2. No  3. I don’t know |
| 45. Do you manage swelling by cold compress? (P) | 1. Yes  2. No  3. I don’t know |
| 46. Do you use medication (analgesics) for swelling and pain after vaccination? (P) | 1. Yes  2. No  3. I don’t know |

…………………….. ………………………. …………………….

Signature of the Signature of the Signature of the

participant interviewer researcher

Recommendation:
