# Supplementary material for: Factors associated with low childhood immunization coverage among Rohingya refugee parents in Cox’s Bazar, Bangladesh
Source: PLoS One. 2023 Apr 7;18(4):e0283881. doi: 10.1371/journal.pone.0283881 (PMC10081790; doi:10.1371/journal.pone.0283881)
Supplement: S1 Table — (DOCX) [file pone.0283881.s002.docx]

**S1 Table. Knowledge, attitude and practice question scoring scheme**

| **Attribute** | **No** | **Question no** | **Answers** | **Score** |
| --- | --- | --- | --- | --- |
| **Knowledge** | 1 | Do you know that vaccination is very important for children from the first day of birth? | 1. Yes | 1 |
|  |  |  | 2. No | 0 |
|  |  |  | 3. Don’t know | 0 |
|  | 2 | Do you know that vaccination prevent infectious disease? | 1. Yes | 1 |
|  |  |  | 2. No | 0 |
|  |  |  | 3. Don’t know | 0 |
|  | 3 | Do you know that vaccination decreases the rates of mortality and disabilities? | 1. Yes | 1 |
|  |  |  | 2. No | 0 |
|  |  |  | 3. Don’t know | 0 |
|  | 4 | Do you know that vaccination could maintain child health? | 1. Yes | 1 |
|  |  |  | 2. No | 0 |
|  |  |  | 3. Don’t know | 0 |
|  | 5 | Do you know that childhood vaccines could control Measles? | 1. Yes | 1 |
|  |  |  | 2. No | 0 |
|  |  |  | 3. Don’t know | 0 |
|  | 6 | Do you know that hepatitis B virus could be prevented by vaccination? | 1. Yes | 1 |
|  |  |  | 2. No | 0 |
|  |  |  | 3. Don’t know | 0 |
|  | 7 | Do you know that diphtheria, tetanus and pertussis could be controlled by vaccination? | 1. Yes | 1 |
|  |  |  | 2. No | 0 |
|  |  |  | 3. Don’t know | 0 |
|  | 8 | Do you know that malnutrition, low fever and diarrhea are not contraindications for vaccination? | 1. Yes | 1 |
|  |  |  | 2. No | 0 |
|  |  |  | 3. Don’t know | 0 |
|  | 9 | Do you know that even healthy child needs vaccination? | 1. Yes | 1 |
|  |  |  | 2. No | 0 |
|  |  |  | 3. Don’t know | 0 |
|  | 10 | Do you know that vaccination could result in skin rash? | 1. Yes | 1 |
|  |  |  | 2. No | 0 |
|  |  |  | 3. Don’t know | 0 |
|  |  |  |  |  |
| **Attitude** | 1 | What do you think about vaccination benefits? | 1. Beneficial | 3 |
|  |  |  | 2. Not beneficial | 1 |
|  |  |  | 3. I don’t know | 2 |
|  | 2 | What do you feel when vaccinating your child? | 1. Safe | 3 |
|  |  |  | 2. Fear | 1 |
|  |  |  | 3. I don’t know | 2 |
|  | 3 | Are you in favor of obligatory vaccination programs designed by the health authorities? | 1. Yes | 3 |
|  |  |  | 2. No | 1 |
|  |  |  | 3. I don’t know | 2 |
|  | 4 | Will you give advice your relatives and family to immunize their children? | 1. Yes | 3 |
|  |  |  | 2. No | 1 |
|  |  |  | 3. I don’t know | 2 |
|  |  |  |  |  |
| **Practice** | 1 | Information on whether the child/children received EPI scheduled vaccines timely (confirmed by seeing the vaccination card from the participant)? | 1. Yes | 1 |
|  |  |  | 2. No | 0 |
