# Supplementary material for: Factors associated with low childhood immunization coverage among Rohingya refugee parents in Cox’s Bazar, Bangladesh
Source: PLoS One. 2023 Apr 7;18(4):e0283881. doi: 10.1371/journal.pone.0283881 (PMC10081790; doi:10.1371/journal.pone.0283881)
Supplement: S2 Table — (DOCX) [file pone.0283881.s003.docx]

**S2 Table. Distribution of responses to knowledge (K), attitude (A) and practice (P) and related questions**

| **Attribute** | **No** | **Question no** | **Answers** | **n (%)** |
| --- | --- | --- | --- | --- |
| **Knowledge** | 1 | Do you know that vaccination is very important for children from the first day of birth? | 1. Yes | 238 (97.5) |
|  |  |  | 2. No | 6 (2.5) |
|  | 2 | Do you know that vaccination prevent infectious disease? | 1. Yes | 221 (90.6) |
|  |  |  | 2. No | 23 (9.4) |
|  | 3 | Do you know that vaccination decreases the rates of mortality and disabilities? | 1. Yes | 208 (85.2) |
|  |  |  | 2. No | 36 (14.8) |
|  | 4 | Do you know that vaccination could maintain child health? | 1. Yes | 233 (95.5) |
|  |  |  | 2. No | 11 (4.5) |
|  | 5 | Do you know that childhood vaccines could control Measles? | 1. Yes | 226 (92.6) |
|  |  |  | 2. No | 18 (7.4) |
|  | 6 | Do you know that hepatitis B virus could be prevented by vaccination? | 1. Yes | 226 (92.6) |
|  |  |  | 2. No | 18 (7.4) |
|  | 7 | Do you know that diphtheria, tetanus and pertussis could be controlled by vaccination? | 1. Yes | 162 (66.4) |
|  |  |  | 2. No | 82 (33.6) |
|  | 8 | Do you know that malnutrition, low fever and diarrhea are not contraindications for vaccination? | 1. Yes | 183 (75.0) |
|  |  |  | 2. No | 61 (25.0) |
|  | 9 | Do you know that even healthy child needs vaccination? | 1. Yes | 238 (97.5) |
|  |  |  | 2. No | 6 (2.5) |
|  | 10 | Do you know that vaccination could result in skin rash? | 1. Yes | 198 (81.1) |
|  |  |  | 2. No | 46 (18.9) |
|  |  |  |  |  |
| **Attitude** | 1 | What do you think about vaccination benefits? (A) | 1. Beneficial | 240 (98.4) |
|  |  |  | 2. Not beneficial | 0 |
|  |  |  | 3. I don’t know | 4 (1.6) |
|  | 2 | What do you feel when vaccinating your child? (A) | 1. Safe | 234 (95.9) |
|  |  |  | 2. Fear | 10 (4.1) |
|  |  |  | 3. I don’t know | 0 |
|  | 3 | Are you in favor of obligatory vaccination programs designed by the health authorities? (A) | 1. Yes | 231 (94.7) |
|  |  |  | 2. No | 1 (0.4) |
|  |  |  | 3. I don’t know | 12 (4.9) |
|  | 4 | Will you give advice your relatives and family to immunize their children? (A) | 1. Yes | 242 (99.2) |
|  |  |  | 2. No | 1 (0.4) |
|  |  |  | 3. I don’t know | 1 (0.4) |
|  |  |  |  |  |
| **Practice** | 1 | Information on whether the child/children received EPI scheduled vaccines timely (confirmed by seeing the vaccination card from the participant)? | 1. Yes | 154 (63.1) |
|  |  |  | 2. No | 90 (36.9) |
